# The complete chloroplast genome of *Cymbidium changningense* (Orchidaceae)

**DOI:** 10.1080/23802359.2019.1688107

**Published:** 2019-11-12

**Authors:** Fang Zheng, Gui-Zhen Chen, Ting-Zhang Li, Zhi-Cong Huang, Meng Wang

**Affiliations:** a Key Laboratory of National Forestry and Grassland Administration for Orchid Conservation and Utilization, Shenzhen, China;; b Shenzhen Key Laboratory for Orchid Conservation and Utilization, The National Orchid Conservation Centre of China and The Orchid Conservation and Research Centre of Shenzhen, Shenzhen, China

**Keywords:** *Cymbidium changningense*, chloroplast genome, phylogenetic analysis, *Cymbidium*

## Abstract

*Cymbidium changningense* is an ornamental orchid and endemic specie in China. Here, we report the complete chloroplast (cp) genome sequence and the cp genome features of *C. changningense.* The cp genome was 155,388 bp in length with a typical quadripartite structure, which was comprised of one large single copy (LSC, 84,522 bp) region and one small single copy (SSC, 20,622 bp) region separated by two inverted repeat (IR, 25,122 bp) regions. The cp genome encoded 132 genes, of which 108 were unique genes (80 protein-coding genes, 24 tRNAs, and four rRNAs). The phylogenetic analysis showed that *C. changningense* was sister with *C. erythraeum.*

*Cymbidium changningense* Z.J.Liu & S.C.Chen has been cultivated in lower reaches of Lancang River for years, where it is called ‘white swan’ (Liu et al. 2005). The plant is well-known for its ornamental flowers, and it is an epiphytic or lithophytic orchid in the genus *Cymbidium* (Swartz 1799, p. 6), which is native of tropical and subtropical Asia, south to Papua New Guinea and North-Australia (Chen et al. 2009; Pridgeon et al. 2009), usually growing in cooler climates at high elevation. Currently, some generic delimitations and infrageneric systems have been proposed in this genus (Schlechter 1924; Hunt 1970; Seth and Cribb 1984; Puy and Cribb 1988; Liu et al. 2006; Chen et al. 2009).

So far, about 16 complete chloroplast (cp) genome sequences of *Cymbidium* have been reported, such as *C. erythraeum* (Huang et al. 2019), *C. floribundum* (Zhang et al. 2019), and *C. mastersii* (Zheng et al. 2019). The data of complete cp genome will serve as a foundation to species identification, germplasm diversity, genetic engineering of *Cymbidium*. We reported the complete cp genome sequence of *C. changningense* in this study.

Leaf samples of *C. changningense* were obtained from the Orchid Conservation and Research Centre of Shenzhen, and specimens were deposited in the National Orchid Conservation Centre (NOCC; specimen code Z.J.Liu 6430) herbarium. Total genomic DNA was extracted from fresh material using the modified CTAB procedure of Doyle and Doyle (1987). Sequenced on Illumina HiSeq 2500 platform (San Diego, CA). Genome sequences was screened out and assembled with MITObim v1.8 (Hahn et al. 2013), which resulted in a complete circular sequence of 155,388 bp in length. The cp genome was annotated with CpGAVAS (Liu et al. 2012).

The cp genome sequence of *C. changningense* (GenBank accession MK848044) was 155,388 bp in length with a typical quadripartite structure, which was comprised of one large single copy (LSC, 84,522 bp) region and one small single copy (SSC, 20,622 bp) region separated by two inverted repeat (IR, 25,122 bp) regions. The cp genome encoded 132 genes, of which 108 were unique genes (80 protein-coding genes, 24 tRNAs, and four rRNAs).

To confirm the phylogenetic position of *C. changningense*, 17 species from the genus *Cymbidium* were included in the phylogenetic analysis. *Pleione formosana* and *P. bulbocodioides* were used to root the tree. The sequences were aligned using MAFFT v7.307 (Katoh and Standley 2013); maximum-likelihood (ML) analysis was conducted using the RAxML software (Stamatakis 2014) with 1000 bootstrap replicates. The phylogenetic tree revealed that *C. changningense* was most related taxa with *C. erythraeum* and then nested in the other *Cymbidium* species (Figure 1). This report will open up avenues for further research to understand the genomic information of the cps of *Cymbidium* and further study on application in phylogeny, species identification and genetic engineering.

**Figure 1.**
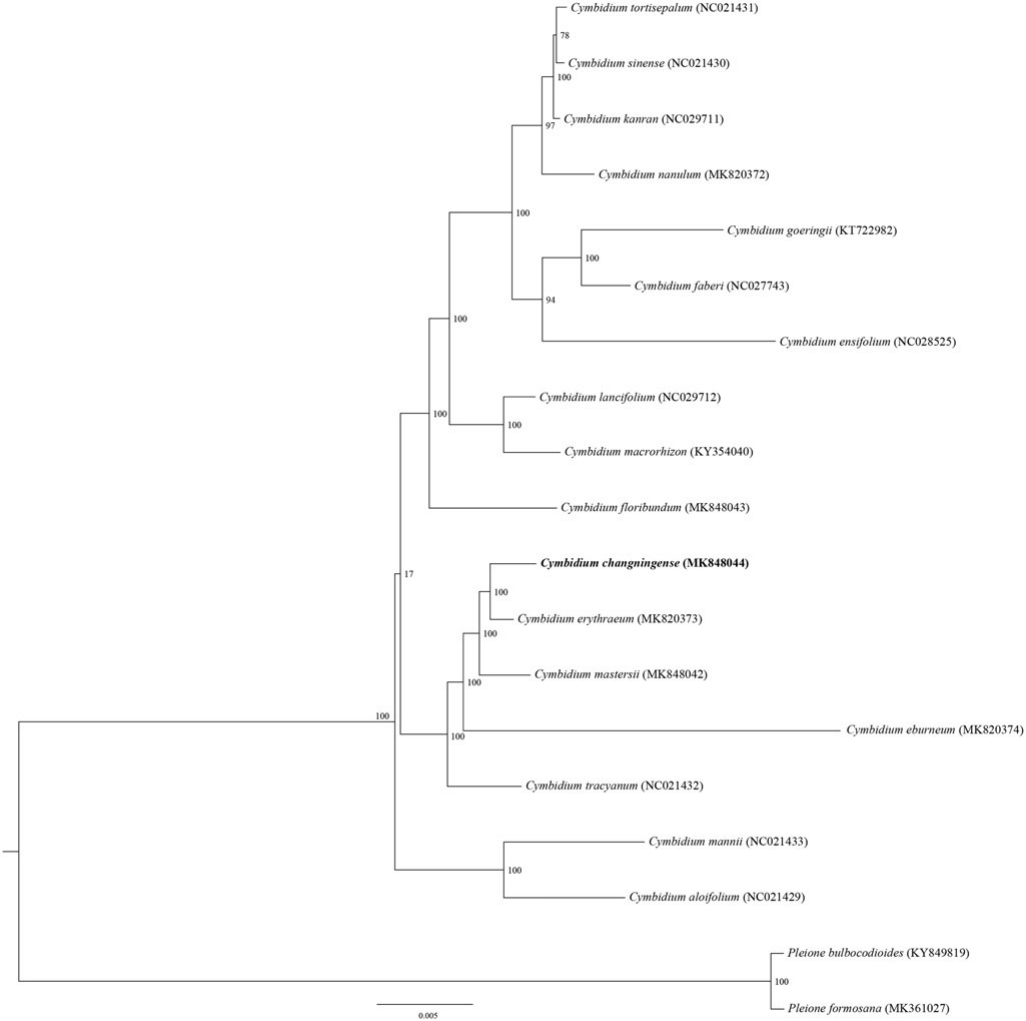
Phylogenetic position of *Cymbidium changningense* inferred by maximum-likelihood (ML) of complete cp genome. The bootstrap values are shown next to the nodes.
